# Formation and structural features of micelles formed by surfactin homologues

**DOI:** 10.3389/fbioe.2023.1211319

**Published:** 2023-07-07

**Authors:** Michał Bochynek, Agnieszka Lewińska, Maciej Witwicki, Agnieszka Dębczak, Marcin Łukaszewicz

**Affiliations:** ^1^ Department of Biotransformation, Faculty of Biotechnology, University of Wroclaw, Wroclaw, Poland; ^2^ InventionBio S.A., Bydgoszcz, Poland; ^3^ Faculty of Chemistry, University of Wroclaw, Wroclaw, Poland; ^4^ OnlyBio S.A., Bydgoszcz, Poland; ^5^ Łukasiewicz Research Network—New Chemical Syntheses Institute, Puławy, Poland

**Keywords:** surfactin, *Bacillus subtilis*, homologues, micelles, adsorption, aggregation number

## Abstract

Surfactin, a group of cyclic lipopeptides produced by *Bacillus subtilis*, possesses surfactant properties and is a promising natural and biologically active compound. In this study, we present a comprehensive characterization of surfactin, including its production, chromatographic separation into pure homologues (C_12_, C_13_, C_14_, C_15_), and investigation of their physicochemical properties. We determined adsorption isotherms and interpreted them using the Gibbs adsorption equation, revealing that the C_15_ homologue exhibited the strongest surface tension reduction (27.5 mN/m), while surface activity decreased with decreasing carbon chain length (32.2 mN/m for C_12_). Critical micelle concentration (*CMC*) were also determined, showing a decrease in *CMC* values from 0.35 mM for C_12_ to 0.08 mM for C_15_. We employed dynamic light scattering (DLS), transmission electron microscopy (TEM), and density functional theory (DFT) calculations to estimate the size of micellar aggregates, which increased with longer carbon chains, ranging from 4.7 nm for C_12_ to 5.7 nm for C_15_. Furthermore, aggregation numbers were determined, revealing the number of molecules in a micelle. Contact angles and emulsification indexes (E_24_) were measured to assess the functional properties of the homologues, showing that wettability increased with chain length up to C_14_, which is intriguing as C_14_ is the most abundant homologue. Our findings highlight the relationship between the structure and properties of surfactin, providing valuable insights for understanding its biological significance and potential applications in various industries. Moreover, the methodology developed in this study can be readily applied to other cyclic lipopeptides, facilitating a better understanding of their structure-properties relationship.

## 1 Introduction

Microbially derived compounds which possess hydrophilic and hydrophobic moieties, and exhibit surface active properties, are commonly referred to as biosurfactants. Compared to chemically derived surfactants, biosurfactants are independent of mineral oil as a feedstock. They are readily biodegradable and can be produced at lower temperatures. Additionally, increasing environmental awareness causes research to be undertaken to produce biosurfactants from biodegradable by-products. Surfactin (SU), which is produced by *Bacillus subtilis*, is becoming one of the best-known biosurfactants. A great deal of previous research into SU has focused on its antifungal, antibacterial, antiviral, and antitumor properties (Vollenbroich et al., 1997; Wen et al., 2011; Wu et al., 2017; Horng et al., 2019) which result from its interaction with biological membranes caused by amphiphilic structure (Seydlová and Svobodová, 2008). However, because of this amphiphilic structure, SU also has interesting aggregation properties and thus can be used as a drug carrier, as demonstrated by our research group (Lewińska et al., 2020; Lewińska et al., 2021; Lewińska et al. 2022). Furthermore, the SU built into the colloidal systems shows synergism; is not only an interfacial stabilizer but also exhibits the properties of an active substance, e.g., antioxidant properties (Lewińska et al., 2021). Many publications demonstrated that SU is a promising agent in the removal of environmental pollutants, for example, in wastewater treatment or oil spillage neutralization (Singh and Cameotra, 2013; Mnif et al., 2015; Ndlovu et al., 2016). Its emulsifier properties enhance the bioavailability of oil ingredients, such as saturated hydrocarbons or polycyclic aromatic hydrocarbons, which then can be degraded by bacterial enzymes (Morán, 2000; Bezza and Chirwa, 2015; Parthipan et al., 2017). Besides its biological activities, cosmetic and pharmaceutical use, SU is also a high utility value molecule.

SU is a collective term used for a group of cyclic lipopeptides (CLP) with a ring composed of seven amino acids that is closed with the *β*-hydroxy-fatty acid moiety (Jajor et al., 2016). Like all CLPs, SU is produced in nonribosomal peptide synthesis (NRPS) from L-amino acids, D-amino acids and fatty acids (Bruner et al., 2002). Because of the substrate variety, structural differences may occur. Surfactin varies in the branching and length of the hydrophobic fatty acid chain. Three isomers, that is *n-*, *iso-* and *anteiso*-, and the lengths of 11–18 carbon atoms have been reported, but only the C_12_-C_17_ homologues have been isolated (Kowall et al., 1998; Bartal et al., 2018; Qin et al., 2023). The most common sequence of the peptide loop is ELLVDLL, in which the second, fourth and seventh residue can be replaced with other amino acids like V, L, I or A. Regardless of the amino acid sequence, their chirality is always preserved (LLDLLDL) (Kowall et al., 1998). In the amino acid ring, two carboxyl groups (E and D) are present, constituting the hydrophilic part of the molecule, while the fatty acid chain forms the hydrophobic tail, to which SU owes its surface activity (surface tension from 72 to 27 mN/m) (Seydlová and Svobodová, 2008).

This publication presents a comprehensive study on the high-yield separation of a surfactin (SU) mixture into individual homologues, with a focus on determining their precise structures and aggregation properties. A wide range of analytical techniques, including chromatography, interfacial tensiometry, dynamic light scattering, transmission electron microscopy, and density functional theory, were employed to thoroughly characterize the homologues. Additionally, the wetting properties and emulsification indexes of the homologue solutions were investigated to gain insights into their dispersing capabilities in water. The findings from this study provide a deeper understanding of the structures and properties of surfactin homologues, with potential applications in diverse fields such as materials science and drug delivery.

## 2 Materials and methods

### 2.1 Materials

LC-MS gradient-grade methanol and acetonitrile were purchased from VWR. Reagent grade salts [NH_4_NO_3_, MgSO_4_, Na_2_SO_4_, KCl, Fe_2_(SO_4_)_3_, CuSO_4_, MnSO_4_], HCl, H_2_SO_4_, and solvents (ethanol, ethyl acetate were purchased from Chempur (Tarnowskie Góry, Poland). Fermentation medium ingredients (yeast extract, MOPS, L-Val, L-Leu, L-Glu) of reagent grade were purchased from BioShop. LC-MS grade HCOOH and phenylthioisocyanate were supplied from Merck. The water used was miliQ using the HLP104V Hydrolab reverse-osmosis system (Wiślina, Poland).

### 2.2 Surfactin production and purification

In order to obtain a mixture of surfactin homologues, the lab-scale bioreactor Labfors3 (Infors AG) was used. 1.6 L of Landy medium (Jajor et al., 2016) (glucose 60 g/L, MOPS 21 g/L, NH_4_NO_3_ 2.3 g/L, MgSO_4_ 0.5 g/L, yeast extract 1 g/L, KCl 0.5 g/L, Fe_2_(SO_4_)_3_ 1.2 mg/L, CuSO_4_ 1.6 mg/L, MnSO_4_ 5.6 mg/L and amino acids L-Glu, L-Leu, L-Val each 1 g/L, initial pH 7.5) was inoculated with *B. subtilis* 87Y strain to OD_0_ = 0.1. Submerged fermentation was carried out at 37°C with 3.2 L/min airflow and 750 rpm stirring. After 24 h the process was terminated and the fermented medium was centrifuged (17,000 G, 10 min) in order to remove bacteria cells. Then the pH of the supernatant was adjusted to 2 with 6 M HCl_(aq)_ and left overnight at 4°C. Next day the precipitated SU was centrifuged (17,000 G, 10 min) and lyophilized. Dry solid residue was then suspended in ethyl acetate, filtered through a short silica plug and dried under N_2_ flow. The obtained SU was used for flash chromatography separation.

### 2.3 Flash chromatography

The surfactin mixture was separated into pure homologues with the use of a reversed phase flash column (Biotage^®^ Sfär C18 Duo 100 Å, 30 μm—120 g) attached to Gilson PLC 2050 (pump system equipped with auto-collector). The mobile phase flow rate was set to 50 mL/min and it was composed of 20 mM phosphate buffer (pH = 6.5) and acetonitrile. Gradient time program is given in Table 1. 3.2 g of surfactin mixture was dissolved in 5 mL of initial mobile phase (buffer - MeCN, 3:7, [*v/v*]) and injected with a syringe directly onto the column. Fractions were collected by auto-collector in 25 mL glass tubes with the detector’s threshold set to 200 mAU at wavelength 210 nm. The collected fractions were analyzed with HPLC-UV and those with the same homologues were combined to obtain C_12_, C_13_, C_14,_ and C_15_ surfactin. These combined fractions were evaporated to dryness with a rotary evaporator. Then the solid residue was dissolved in 50 mL of AcOEt, dried with anhydrous Na_2_SO_4_, pushed through a small silica gel plug with ethyl acetate (in order to remove inorganic impurities), filtered with a 0.22 µm nylon syringe filter and dried. The colorless, transparent, glass-like solid was dissolved in 10 mL of EtOH, diluted with the same volume of water, frozen in −80°C and lyophilized to get a product in the form of white powder with more than 99% purity.

**TABLE 1:** Gradient time program for flash separation.

Time [h:min:s]	Phosphate buffer [%]	Acetonitrile [%]
0:00:00	30	70
0:07:00	30	70
0:17:00	38	62
0:20:00	38	62
0:25:00	40	60
1:30:00	40	60
2:10:00	42	58
2:10:00	42	58
2:25:00	55	45

### 2.4 HPLC-UV analysis of fractions

The fractions collected during flash separation were analyzed with a HPLC-UV system composed of two ECOM ALPHA 10 PLUS solvent pumps and detector UV-VIS EXOM Sapphire 600. The device was equipped with a 20 µL injection loop, KINETEX^®^ 5 μm EVO C18 (100 Å, 150 mm × 4.6 mm) column and controlled by computer with Lp-Chrom^®^ (Lipo-pharm.pl) software. From every 4th glass tube, 200 µL of fraction was diluted fivefold in MeOH and injected for analysis. The mobile phase (consisting of 15% water with 0.1% (*v/v*) HCOOH and 85% MeCN with 0.1% (*v/v*) HCOOH) was pumped with 1 mL/min flow in isocratic mode. 8 min was enough to elute all SU homologues from the column. The fractions containing one pure homologue were combined while the multicomponent fractions were rejected.

### 2.5 LC-MS analysis of surfactin homologues

Analyses were performed on Shimadzu LCMS-8040 system equipped with an ESI ion source and triple quadrupole mass detector. As the stationary phase Cortex C18 column (sizes: 4.6 mm × 50 mm, 2.7 µm) was used. During the experiment, the column was heated to 40°C and samples were kept at 10°C. Mobile phases were MeCN with 0.1% (*v/v*) HCOOH and water with 0.1% (*v/v*) HCOOH. The separation method was 10 min long with 1 mL/min flow rate of mobile phase and injection volume equal to 4 µL. The starting eluent was mixture 1:1 of MeCN and water and during the separation, the acetonitrile content was increasing. For the homologues’ purity assay the analyzed mass range was 990–1,100 m/z in positive ion mode. The ion source temperature was set to 600°C, cone voltage 25 V and capillary voltage 0.8 kV. The desolvation gas was nitrogen. For fragmentation experiments, the collision energies (with argon as the collision gas) were optimized for every homologue in order to obtain the best mass spectrum.

### 2.6 Hydrolysis of surfactin

10 mg of each SU homologue was placed in glass ampoule with 1 mL of 6 M HCl_(aq)_, flushed with gaseous N_2_ and capped by melting the glass. The hydrolysis was conducted for 24 h in 110°C. Then the hydrolysates were transferred to 10 mL glass tubes, diluted with 3 mL portions of distilled water and extracted thrice with 3 mL aliquots of diethyl ether. Organic extracts were combined and evaporated under N_2_ stream to give samples containing *β*-hydroxy-fatty acids. Water layers were lyophilized in order to obtain white, solid residues for amino acids analysis.

### 2.7 GC-MS analysis of fatty acids

Extracted *β*-hydroxy-fatty acids were derivatized in order to obtain methyl esters (You et al., 2015). Each sample was combined with 1 mL of 10% H_2_SO_4_ in MeOH, vortexed and incubated for 6 h in 55°C. Then after cooling down esters were extracted with diethyl ether (3 mL × 3 mL), dried and dissolved in 1 mL of methanol to be analyzed on Shimadzu GC-MS system (Shimadzu GC-MS TQ 8040) equipped with Zebron ZB-5MSi capillary column (30 m × 0.32 mm × 0.25 µm) with helium as a carrier gas (flow rate set to 1 mL/min). The column temperature was initially held at 60°C for 3 min, then elevated to 260°C at a rate of 8°C/min and maintained for 10 min. The EI ion source temperature was set at 220°C with 70 eV of ionization energy. The injection was performed at 250°C with sample volume equal to 5 μL at split ratio 20:1. The results were compared with the NIST11 Database.

### 2.8 HPLC-UV analysis of fatty acids

Amino acids were derivatized with phenylisothiocyanate (PITC) in order to obtain UV-absorbing products separatable on C_18_ column (Gonzalez-Castro et al., 1997). 0.3 mg of each sample isolated after hydrolysis was placed in glass vial and dissolved with 50 µL of redrying mixture (methanol-water-triethylamine 2:1:1 [*v/v*]). Solvents were removed under vacuum pump (160–180 mbar) and solid residue was mixed with 30 µL of derivatizing agent (methanol-water-triethylamine-PITC 7:1:1:1 [*v/v/v/v*]). After vortexing reaction was left for 20 min at room temperature and then placed under vacuum (160–180 mbar) for 4 h in order to remove solvents and derivatization byproducts. Dried samples were suspended in 300 µL of injection mixture (0.1 M phosphate buffer pH = 7.5 with 5% acetonitrile). Suspension was vortexed, agitated in ultrasound bath and centrifuged at 9050 G for 4 min before injection (10 µL). Analyzes were performed on ArcAcquity HPLC-UV system (Waters) equipped with Waters Pico-Tag column (300 mm × 3.9 mm; 4 µm) held at constant temperature 30°C. Eluent A was an aqueous 0.14 M sodium acetate with addition of triethylamine (0.5 mL/L) and pH set to 6.2 with glacial acetic acid. Eluent B was acetonitrile-water (40:60 [*v/v*]). The mobile phase flow and gradient were taken from (Gonzalez-Castro et al., 1997). Measured results were compared to the standards: L-Glu (E), L-Asp (D), L-Val (V), L-Leu (L) and L-Ile (I). The UV detector was set to 254 nm which is wavelength in absorption band of PITC phenyl ring.

### 2.9 Gibbs isotherms measurement

The surface tension measurements were performed at 295 ± 0.1 K using a Krüss K12 microprocessor tensiometer (Krüss, Hamburg, Germany) equipped with a du Nouy Pt-Ir ring (resolution ±0.01 mN/m). The surface tension data were obtained as the arithmetic mean of the values received from two independent runs; the data were reproducible within ±0.2 mN/m. From the data, the values of adsorption properties were calculated.

### 2.10 DLS measurement

Measurements were conducted at 298 K, using a Zetaseizer Nano Series (Malvern Instruments) equipped with ALV5000 multi-τ autocorrelator and He-Ne laser (632.8 nm) as a light source. The detection angle was set to 173° and samples were placed in disposable folded capillary cells (DTS1070) produced by Malvern Instruments. Each SU homologue was prepared as a solution of concentration equal to 10 × *CMC*, filtered through a 0.22 µm nylon syringe filter, placed in the measurement cell and left overnight to equilibrate before measurement. Analyses were performed in three runs and one run consisted of at least 10 measurements, and the obtained data is expressed as an average quantity with standard deviation. Due to the low solubility of pure SU in water, all measurements were done in 0.05 M TRIS×HCl (pH = 8.5).

### 2.11 TEM imaging

The transmission electron microscopy (TEM) imaging was performed using a FEI Tecnai G2 XTWIN transmission electron microscope (FEI, Hillsboro, OR, United States. The samples were prepared by placing a small amount of diluted suspension on a Cu-Ni grid and stained with 2% uranyl acetate before shooting. The size distribution plots were fitted using a Gaussian curve approximation.

### 2.12 DFT calculations

Computational techniques based on density functional theory (DFT) have been an effective tool for studying various chemical, biochemical or environmental problems (Ćwieląg-Piasecka et al., 2017; Rodríguez-Blanco et al., 2020; Witwicki et al., 2020; Witwicki et al., 2021; Cao et al., 2022; DeepikaVerma et al., 2022; Gorb et al., 2022) In our work, DFT calculations were conducted with the ORCA 5 suite of programs (Neese, 2012; Neese et al., 2020; Neese, 2022). In all DFT calculations, the resolution of the identity approximation (Neese, 2003; Kossmann and Neese, 2009; Neese et al., 2009), the dispersion correction with the Becke-Johnson damping scheme (D3BJ) (Grimme et al., 2010; Grimme et al., 2011), the conductor-like polarizable continuum model (CPCM) (Barone and Cossi, 1998) to cover solvent (water) effects, and the def2 basis sets (Weigend and Ahlrichs, 2005) in combination with the appropriate auxiliary basis set (def2/J) (Weigend, 2006) were used. Initial structures for geometry optimizations were generated using the genetic algorithm with the initial population of 200 conformers as implemented in OpenBabel 2.4.0 (O’Boyle et al., 2011). The geometry optimizations were carried out using the gradient-corrected functional BP86 (Perdew, 1986; Becke, 1988), which provides accurate molecular structures (Neese et al., 2010; Witwicki, 2015), the basis set def2-SVP, the tight SCF convergence criteria (TightSCF) and the default scheme for numerical integration (DefGrid2). Each of the stationary points was fully characterized as a true minimum through a vibrational analysis. On these molecular structures, single-point calculations were conducted with the hybrid functional B3LYP (Lee et al., 1988; Becke, 1993; Stephens et al., 1994), the basis set def2-TZVP, tightened SCF convergence criteria (VeryTightSCF) and increased accuracy of numerical integration (DefGrid3).

Electron density from these single-point calculations was analyzed with the Multiwfn 3.8dev code (Lu and Chen, 2012a). The electrostatic potential (EP) was studied on the van der Waals (vdW) surface defined as an electron density equal to 0.001 au. Such an approach to EP has been demonstrated to reflect accurately the possible electrostatic interaction between a molecule and other molecules (Bader et al., 1987; Lu and Chen, 2012b; Liu et al., 2021). Quantitative molecular surface analysis was carried out using the improved Marching Tetrahedra algorithm (Liu et al., 2021). EP was evaluated by regrouping the expression in terms of primitive Gaussian orbitals with identical angular momentum types and nuclei centers (Zhang and Lu, 2021). Molecular polarity index (MPI) was calculated as described in the literature (Liu et al., 2021). Visualizations were done with a combination of Gabedit (Allouche, 2011) and POV-Ray (www.povray.org) software.

### 2.13 Emulsification index

Emulsification indexes were determined with a standard method (Cooper and Goldenberg, 1987), where 6 mL hydrophobic fraction was added to 4 mL of surfactant solution of 150 mg/L concentration. Experiments were carried out in glass tubes with dimensions of 15 mm (internal diameter), 100 mm (length) and glass thickness of 1 mm. The bottoms were rounded and tubes were screw capped. After mixing samples were vortexed for 2 min at maximum speed and left to equilibrate for 24 h. After 24 h, the heights of the layers were measured (oil, emulsion, water and overall). In order to calculate E_24_, the height of the emulsion layer was divided by the overall sample’s height and expressed in percent:

$$E_{24}=\frac{h_{emulsion}}{h_{overall}}*100\%$$ (1)

### 2.14 Contact angle assay

All samples were analyzed with the use of two different surfaces, glass (hydrophilic) and plastic - polyethylene (hydrophobic). In the experiments, solutions with 0.11 mg/mL concentrations of pure homologues in 0.05 M TRIS×HCl buffer (pH = 8.5) were prepared and placed on the surface as one droplet. Contact angles were measured with Kruss DSA 100 in standard conditions (273 K, 1,013 hPa) for hanging droplets.

## 3 Results and discussion

### 3.1 Flash chromatography

**FIGURE 1:**
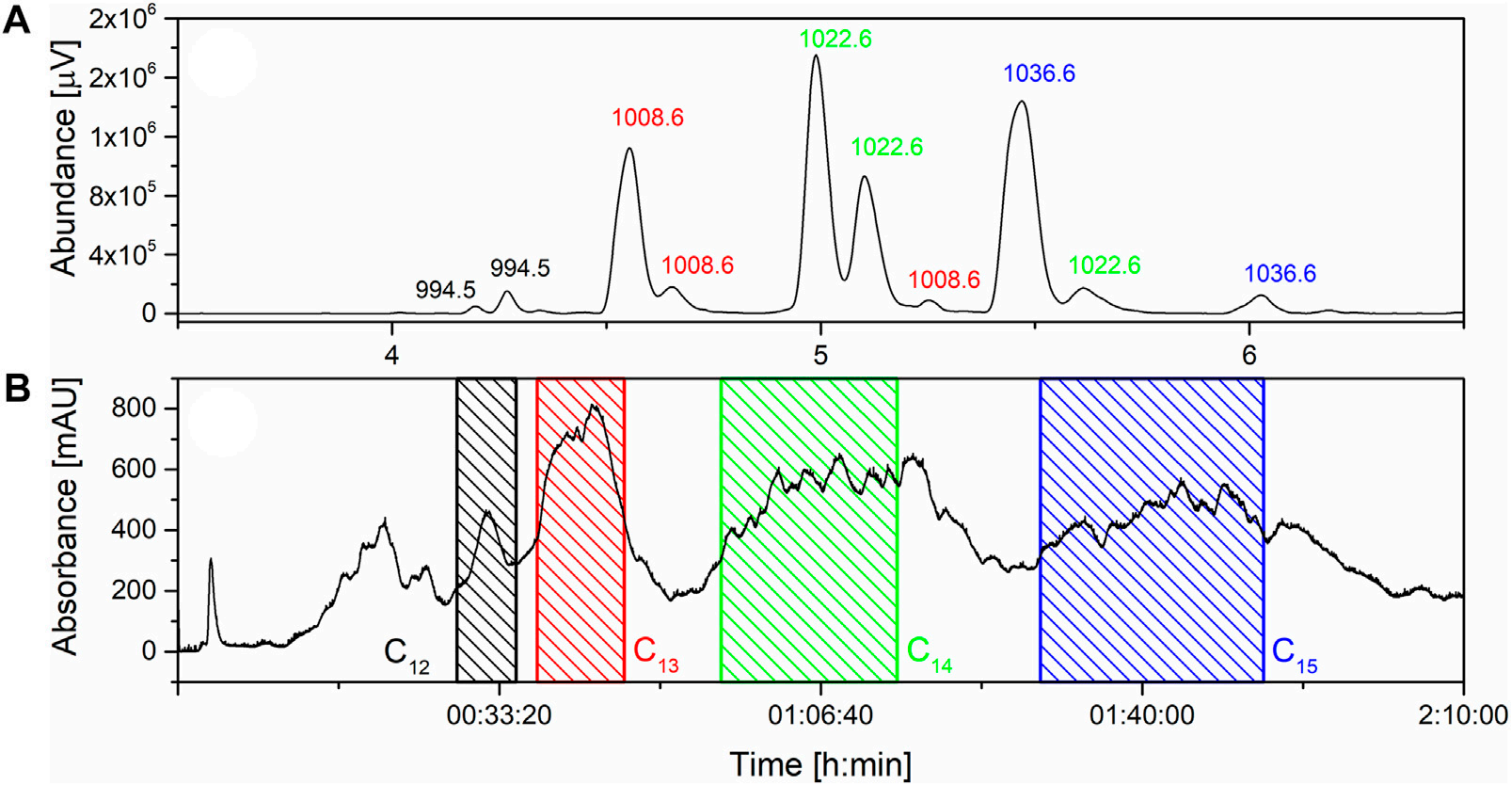
**(A)** composition of surfactin’s mixture with m/z values; **(B)** Flash separation chromatogram with marked fraction collection.

SU derived from microbial fermentation is a mixture of homologues (Figure 1A) which is typically purified via preparative-HPLC to obtain pure homologues with low yields (Peypoux et al., 1991; Kowall et al., 1998; Kracht et al., 1999; Kim et al., 2009). The first trials with flash chromatography involved a water-acetonitrile system as a mobile phase and C_18_ resin with particles sizes 100Å and 30 µM. However, due to the low solubility of pure SU in water dissolving even 1 g of analyte in 5 mL of initial mobile phase was impossible. For this reason, the water was replaced with a phosphate buffer with a concentration of 20 mM and pH stabilized at 6.5. This solution allowed to dissolve more than 3 g of SU in the injected volume (5 mL). First, the separations were tested in an isocratic mode with different mobile phase composition ratios. This approach turned out to be ineffective due to the very long separation time. Therefore, the method was modified and a gradient was introduced in which the concentration of acetonitrile increases with time starting from initial 30% (*v/v*). The gradient was optimized by multiple attempts to separate the SU into homologues shown in the chromatogram (Figure 1B). Using a column containing 120 g of C_18_ resin, it is possible to inject 3.2 g of SU in 5 mL of initial mobile phase and, as a result, isolate pure homologues C_13_, C_14_ and C_15_ in amounts exceeding 100 mg. In addition, it is possible to isolate the C_12_ homologue, but in much smaller amounts due to its low content in the injected sample. Isolated fractions were analyzed with HPLC-UV apparatus in order to determine their compositions. All fractions containing pure homologues were combined and subjected to further isolation from mobile phase (described in materials and methods). Finally, homologues with over 99% purity were obtained. To our knowledge, this method is the most efficient way to isolate pure SU homologues enabling physiochemical and biological characterization (Peypoux et al., 1991; Kowall et al., 1998; Kracht et al., 1999; Kim et al., 2009).

### 3.2 Structural analysis of isolated homologues

**FIGURE 2:**
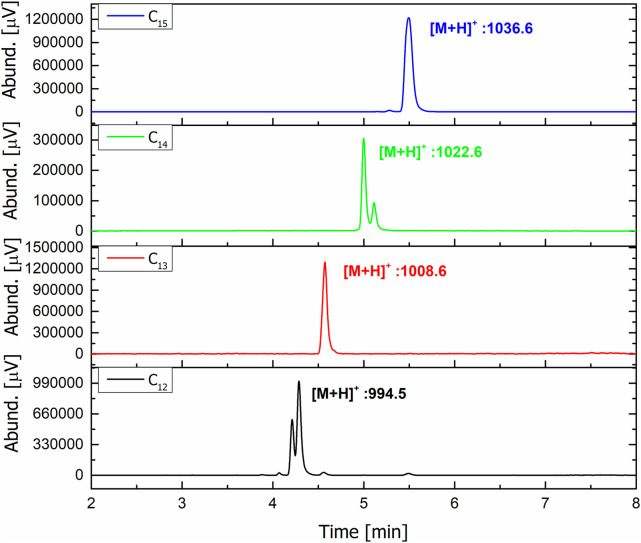
LC-MS scan mode chromatograms of purified fractions.

SU compounds are a group of molecules that exhibit structural variability. First, they can form homologous series due to differences in the number of carbon atoms in *β*-hydroxy-fatty acid chains. Additionally, particular homologues can form isomers, as fatty acid moieties can exist in three branching patterns (*n-, iso*-, and *anteis*o-). Finally, analogues of surfactin can be distinguished based on differences in the amino acid substitution within the heptapeptide ring. Given the above facts, determining the exact structure of the particular SU homologue is not an easy challenge. Attempts similar to ours have been made in the past by, for example, (Grangemard et al., 1997) who characterized new variants of this compound. Their strategy was based on use of the NMR for *β*-hydroxy fatty acid branching determination, GC-MS in order to obtain information about its length and HPLC-UV for measurement of amino acids composition (Arutchelvi et al., 2009). have determined structures of homologues based on the HPLC-UV of amino acids and LC-MS measurement of molar masses but their strategy do not recognize the difference between homologue C_12_ with ELLLDLL and C_13_ ELLVDLL which have the same masses. To fully elucidate the structures of the isolated homologues, we initially assessed their purity using LC-MS in positive ion scanning mode, within the mass-to-charge ratio (m/z) range of 990–1,100 (Figure 2). In an electrospray ion source, surfactin gives adducts with protons [M + H]^+^, sodium cations [M + Na]^+^ and potassium cations [M + K]^+^, so the expected m/z values of all homologues (listed in Table 2) are covered by the measured range (Tang et al., 2010; Bartal et al., 2018; Sun et al., 2019). The results proved high purity of the isolated surfactin homologues, shown by the pseudomolecular ions’ masses. In the cases of C_13_ and C_15_, single peaks were observed, but further analyses (GC-MS) have shown that their asymmetric shapes are caused by the overlapping of peaks indicating two different isomers. For C_12_ and C_14_, mixtures of two isomers were measured–pair of peaks with the same m/z (994.5 for C_12_ and 1022.6 for C_14_).

**TABLE 2:** Surfactin homologues molecular masses and m/z values of pseudomolecular ions.

Homologue	Molecular mass [Da]	m/z for [M + H]^+^	m/z for [M + Na]^+^	m/z for [M + K]^+^
C_12_	993.5	994.5	1,016.5	1,032.5
C_13_	1,007.6	1,008.6	1,030.6	1,046.6
C_14_	1,021.6	1,022.6	1,044.6	1,060.6
C_15_	1,035.6	1,036.6	1,058.6	1,074.6

**FIGURE 3:**
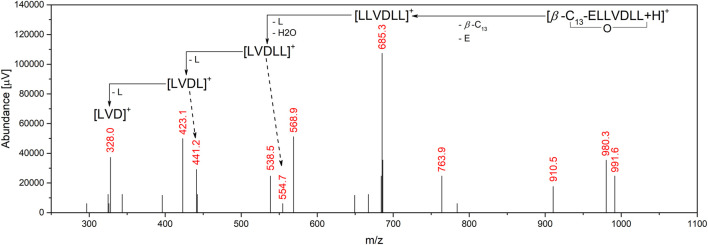
Fragmentation pattern observed on MS spectrum.

In most cases of surfactin production, the sequence of peptide ring is the same for all homologues produced in one batch, but there are some examples in literature showing that, for example, leucine (L) from 2nd position can be replaced with isoleucine (I), valine (V) from 4th position can be replaced with L or alanine (A), and L from 7th position can be replaced with I or V (Bartal et al., 2018; Hu et al., 2019). Therefore, to determine the amino acid sequences, MS/MS experiments were done. The first step was the selection of proper collision energies to obtain the best fragmentation patterns. It turned out that the longer the homologue, the higher the collision energy necessary, and finally −35 V was used for C_12_ and C_13_, and -40 V for C_14_ and C_15_ homologues. In all cases, the following m/z fragmentation pattern was observed: 685.2 > 554.2 > 441.2 > 328.4. The m/z = 685.2 is the product of the ring opening and cleavage of the *β*-hydroxy-fatty acid and the glutamic acid (E), so the resulting ion is [LLVDLL]^+^. Then, after the loss of the water molecule and N-terminal L, the [LVDLL]^+^ ion is formed (m/z = 554.2). Removal of the C-terminal L gives the [LLVDL]^+^ ion with m/z = 441.2. The last fragmentation reaction involves cleavage of the N-terminal L, resulting in the formation of an ion with m/z = 328.2 (Figure 3) (Ishigami et al., 1995; Tang et al., 2010). Such a fragmentation pattern was observed for all the analyzed homologues, indicating that the 4th position is V, and the 2nd position is either L or I, as mass spectrometry cannot distinguish between these two amino acids. Therefore, amino acid content analysis using HPLC-UV was performed.

**FIGURE 4:**
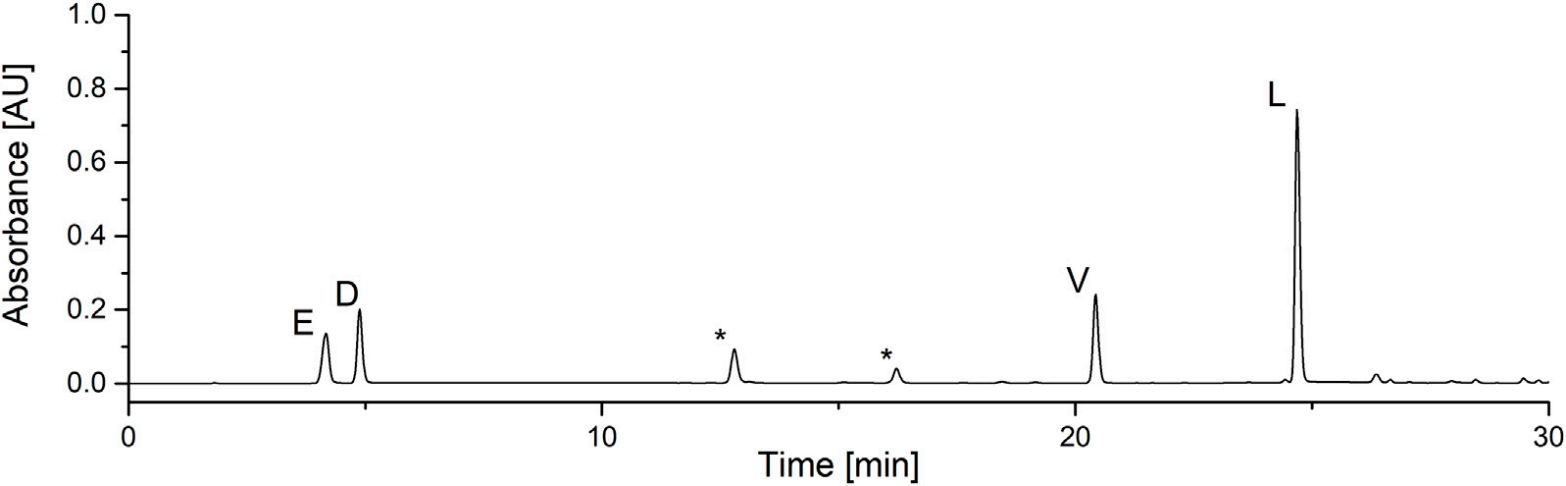
Example HPLC-UV analysis of PITC derivatized hydrolysates (measured for C15).

In order to exclude or prove I presence in the heptapeptide ring of SU’s homologues, samples were hydrolyzed to amino acids and derivatized with the PITC (Bidlingmeyer et al., 1984). HPLC-UV results obtained for amino acid standards provided retention times: 4.20 min – aspartic acid (D), 4.88 min – glutamic acid (E), 20.42 min - valine (V), 24.33 min - isoleucine (I) and 24.70 min - leucine (L). These values compared with chromatograms measured for hydrolyzed homologues have shown an absence of I in all the homologues (chromatogram for C_15_ as an example shown in Figure 4). This means that all analyzed samples have sequence ELLVDLL, and the only structural differences between homologues are *β*-hydroxy-fatty acids. Between E and V peaks there are two non-identified peaks which are impurities (side products from derivatization of D and E, which were also observed in the standards’ chromatograms). Integrals for E and D peaks are much lower than for V due to its weaker detector response factor, which is a known observation (Gonzalez-Castro et al., 1997). For V and L, the detector response factor is almost the same and the ratio between their peaks’ areas is near 1:4, which is consistent with SU’s ring sequence where there are 4 L and 1 V.

**FIGURE 5:**
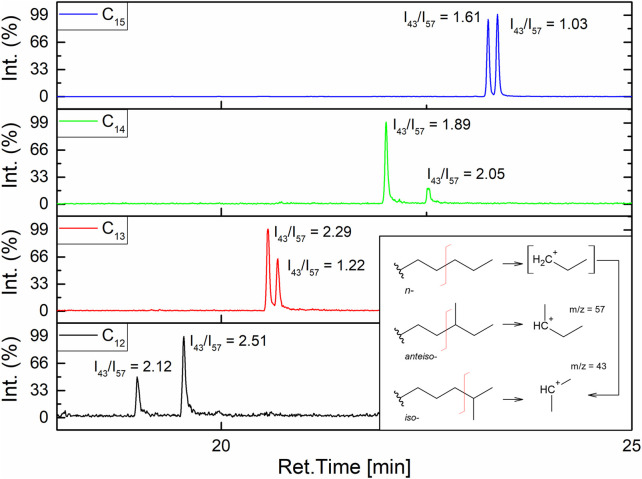
GC-MS analysis of *β*-hydroxy fatty acids in SU’s homologues and calculated I_34_/I_57_ indexes.

To elucidate entire homologues’ structures, *β*-hydroxy-fatty acids’ branching had to be determined. According to the literature, it can be stated that in cases of even numbers of carbon atoms in a homologue, the pair of *n-* and *iso-* isomers is produced. Odd numbered homologues form *iso-* and *anteiso-* pairs, which means that C_12_ has *n-* and *iso-* isomers, and C_15_ has *iso-* and *anteiso-* isomers (Kowall et al., 1998; Zhao et al., 2012) From the GC-MS with EI ion source measurement of fatty acids, pairs of isomers can be distinguished based on the value of I_43_/I_57_ index (Figure 5). The *iso-* chain, as a direct product of fragmentation, forms a secondary carbocation CH_3_^+^CH(CH_3_), and in the case of the *n-*chain, the direct product is a primary carbocation ^+^CH_2_CH_2_CH_3_, which then rearranges to the secondary one (CH_3_^+^CH(CH_3_). Thus, fragmentation to m/z = 43 for *iso-* chain is more preferable than for *n-*. For this reason, *iso-*branched fatty acid gives higher I_43_/I_57_ than *n-*. The *anteiso-* chain gives a direct fragmentation product CH_3_CH_2_^+^CH(CH_3_) with m/z = 57, so its I_43_/I_57_ ratio will be lower than for *iso-*. It means that for the analyzed surfactin’s *β*-hydroxy-fatty acid isomers, this method can be used (Yang et al., 2007). The most stable fragmentation products are presented in Figure 5. GC-MS chromatograms (Figure 5) show two peaks for all homologues, which means that two different isomers are present in all the tested samples. I_43_/I_57_ analysis for the obtained pairs shows that in the case of C_12_ and C_14_, the first peak is a linear chain (*n-*), and the second peak is *iso-*branched because the calculated index for *iso*- is higher than for *n-*. For C_13_ and C_15_, there are *iso*- and *anteiso-* isomers, with the first peak on the GC-MS chromatogram corresponding to the *iso-*branched isomer. It was also observed that the retention times of *iso-* and *anteiso-* isomer pairs vary less compared to *n-* and *iso-* isomer pairs. As a result, the LC-MS chromatograms of *iso-* and *anteiso-* isomer pairs exhibit a single asymmetric peak, in contrast to the two separated peaks observed in the case of *n-* and *iso-* isomer pairs.

### 3.3 Gibbs isotherms measurement

**FIGURE 6:**
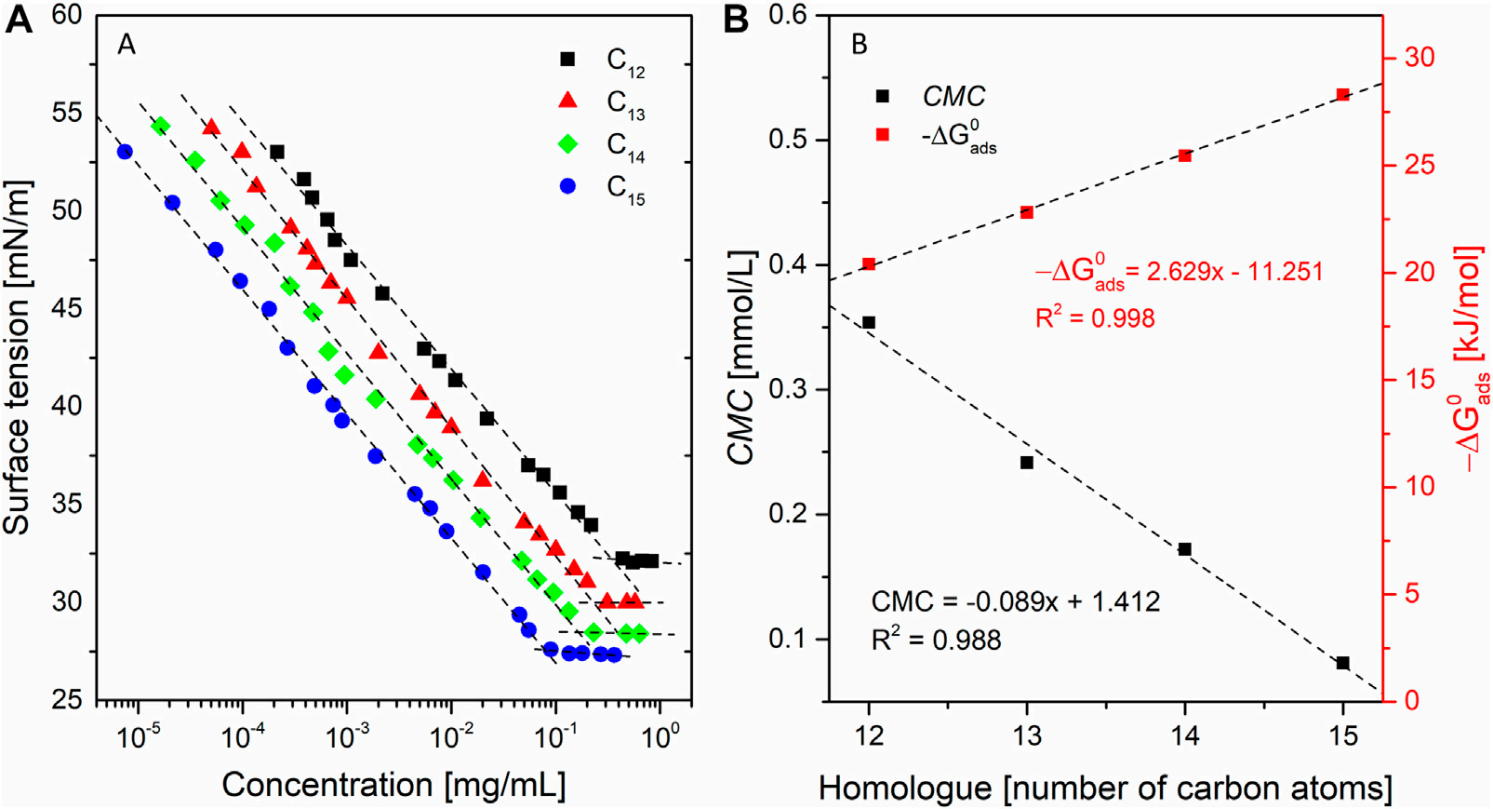
**(A)** equilibrium surface tension (γ) as a function of surfactant concentration; and **(B)** standard free energy of adsorption (∆G^0^_ads_) a critical micelle concentration (CMC), of SU homologues as a function of the number of carbon atoms in the alkyl chain.

The equilibrium surface tension isotherms for solutions of SU homologues in 0.05 M TRIS×HCl buffer (pH = 8.5) are shown in Figure 6A. The interfacial behavior of the SU homologues was studied through surface tension measurements performed at a wide range of surfactant concentrations. The surface tension isotherms and the adsorption parameters derived from the surface tension data were calculated. Classic interfacial behavior was observed as the surface tension decreases as the surfactant concentration increases. Once the minimum is reached, further addition of surfactant no longer changes the surface tension, so that the critical micelle concentration can be determined (Figure 6A).

The determined *CMC*s allow one to calculate the change of standard free energies of micellization (a measure of the tendency to form micelles), and reached 0.35, 0.24, 0.17, 0.08 mmol/L for C_12_, C_13_, C_14_, and C_15_, respectively. In any homologous series of surfactants, *CMC* regularly decreases with increasing hydrocarbon chain length and with increasing surfactant surface activity. This is because of Traube’s rule, which says that in a homologous series of surfactants, each additional methylene group reduces the molar concentration required to reduce the surface tension of water by a factor of three. It means that for diluted solutions of surfactants belonging to one homologous series, in order to obtain the same reduction in the surface tension of the solution, three times lower molar concentration of the surfactant should be used compared to the compound containing one less methylene group in the hydrocarbon chain. It means that adding a methylene group reduces the *CMC* by a factor -log(1/3) = 2. However, deviations from this rule are possible, because it was formulated for simple compounds (such as aliphatic alcohols acids or esters) what should be noted during analysis of macromolecular compounds. Moreover, Traube’s results were approximate since he used not sufficiently low concentrations to obtain linear part of the relation between concentration and surface tension. For the aliphatic alcohols and esters that factor was placed in the range of 2.7–3.2. It was also shown that for longer homologues that factor ranged from 1.2 to 5.8. Further research proved that Traube factor decreases for the compounds of high molecular weights (Traube, 1940). The development of that Traube’s theory with more concentrated solutions gave factors in the wider range 2.5–4.1 (Ward, 1946). Taking into consideration abovementioned deviations it can be stated that our *CMC* values giving factors in pairs C_12_-C_14_ and C_13_-C_15_ equal to 2 and 3 agree with theory. Ignoring the differences in the shapes of the branching patterns obtained factors are: C_12_-C_13_ (1.5) C_13_-C_14_ (1.4), C_14_-C_15_ (2.1). Figure 6B shows how the *CMC* value is influenced by the structure of the surfactant tail (its length)—the longer the alkyl chain, the lower the *CMC* value (Ueno et al., 1981). Determined *CMC* values agrees with the results of similar assays presented in literature (Arutchelvi et al., 2009). determined the *CMC* of crude surfactin isolated from YB7 strain of *B. subtilis* equal to 0.040 mmol/L in water basified with addition of NaOH. That value for mixture of homologues is lower than our determination for C_15_ but there is important difference in the buffering system. *CMC* values of surfactin measured in presence of Na^+^ ions are significantly lower than in their absence (Li, Ye, et al., 2009). Also our not presented results have shown that in 0.01 PBS containing sodium salts *CMC* of C_15_ was measured to 0.020 mmol/L what is 4 times lower value than in 0.05 M TRIS×HCl (Zou et al., 2010). have measured *CMC* 0.015 mmol/L for C_15_ homologue in 0.01 M PBS what agrees our 0.020 mmol/L. Moreover the (Arutchelvi et al., 2009) do not specify the purity of isolated surfactin. The homologues are identified but their structures are not elucidated in exhaustive way. There is no clarity if compound described as C_13_ homologue is C_13_ with ELLDVLL peptide ring sequence of C_12_ with ELLDLLL. Nevertheless, the determined quantity is of the same order of magnitude. Similar results (*CMC* = 50 mmol/L) were published for surfactin standard purchased from Sigma and measured in PBS buffer (Shen et al., 2010). Another example is measurement of *CMC* for C_16_ homologue without the specified *β*-hydroxy fatty acid branching pattern (Li, Ye, et al., 2009). The experiment was conducted in the same buffer as in our assay and the author’s result is 0.025 mmol/L. This is in accordance with Traube’s rule and the tendency of the *CMC* to decrease with the elongation of the homolog molecule (Liu et al., 2008). presented *CMC* value 0.062 mmol/L for C_12_ homologue with the ELLVDLL sequence isolated with 90% purity. The measurement was conducted in 0.01 M PBS so the *CMC* was influenced by Na^+^ ions. Moreover, the chromatogram presented in article suggests presence of longer homologues in the 10% of impurities which may have additionally diminish the *CMC*. The *CMC* for C_17_ homologue measured in 0.01 M PBS by (Qin et al., 2023) was 0.004 mmol/L which also agrees with the Traube rule and the tendency to lower the micellization concentration with addition of following methylene groups and presence of Na^+^ ions. The presence of branches in the chain has an impact on ∆*G* ^*0*^_*ads*_,*,* causing its decrease due to the steric hindrance to aggregate in bulk (Varadaraj et al., 1992). The surface activities, measured as ∆*G* ^*0*^_*ads*_, were obtained from the equation:

$$\Delta G_{ads}^{0}=-0.45\;nC+5.17$$ (2)

Surface activity’s increase with the length of the alkyl chain was observed and its dependence on the number of carbon atoms in the alkyl chain for SU appears to be linear (Figure 6A):

The obtained surface tension data were used to calculate the characteristic adsorption parameters from the Gibbs adsorption equation:

$$\Gamma_{\infty }=-\frac{1}{nRT}\frac{d\gamma }{d\ln_{c}}$$ (3)

where *Γ* [mol/m^2^] is the surfactant surface excess concentration, *γ* [mN/m] is the surface tension, *c* [M] is the surfactant concentration, *R* [J/(mol K)] is the gas constant and *T* [K] is the absolute temperature. The minimum surface area demand per molecule, *A*_min_, was calculated from the equation:

$$A_{\min}=\frac{1}{N_{a}\Gamma_{\infty }}$$ (4)

where *N*_*A*_ is the Avogadro number. The values of the obtained parameters for the investigated surfactants are shown in Table 3. Our results of A_min_ agrees with literature. (Shen et al., 2009) have determined experimentally 147 Å^2^ with SANS and compared it to the Gibbs equation results obtained in non specified buffer system (132 Å^2^) by (Maget-Dana and Ptak, 1995). The values shown increasing tendency with elongation of homologue chain within C_12_,C_14_ and C_15_. The difference between C_12_ and C_13_ is negligible.

$$\Delta G_{ads}^{0}=-2,303\;RT\;pC_{20}$$ (5)

where p*C*_20_ is surface adsorption efficiency as the negative logarithm of bulk surfactant concentration *C*_20_ required to reduce the surface tension of water by 20 mN/m. The more negative the Δ*G* ^0^_ads_ values, the greater the tendency for the surfactant to adsorb at the air/water interface. Thus, the values of Δ*G* ^*0*^_*ads*_ in Table 1 show that an increased length of hydrocarbon chain promotes greater adsorption activity.

**TABLE 3:** Adsorption parameters of surfactin homologues.

*Homologues*	*γ*_min_ [mN/m]	10^6^ *Γ*_*∞*_ [mol/m^2^]	10^20^ *A*_min_ [Å^2^]	p*C*_20_ [M]	-Δ*G* ^0^_ads_ [kJ/mol]	*CMC* [mmol/L]
C_12_	32.2	2.57	149	2.43*10^−4^	20.41	0.35
C_13_	30.0	2.60	147	9.13*10^−5^	22.81	0.24
C_14_	28.5	2.50	153	3.11*10^−5^	25.43	0.17
C_15_	27.6	2.45	156	9.79*10^−6^	28.29	0.08

### 3.4 DLS measurement

**FIGURE 7:**
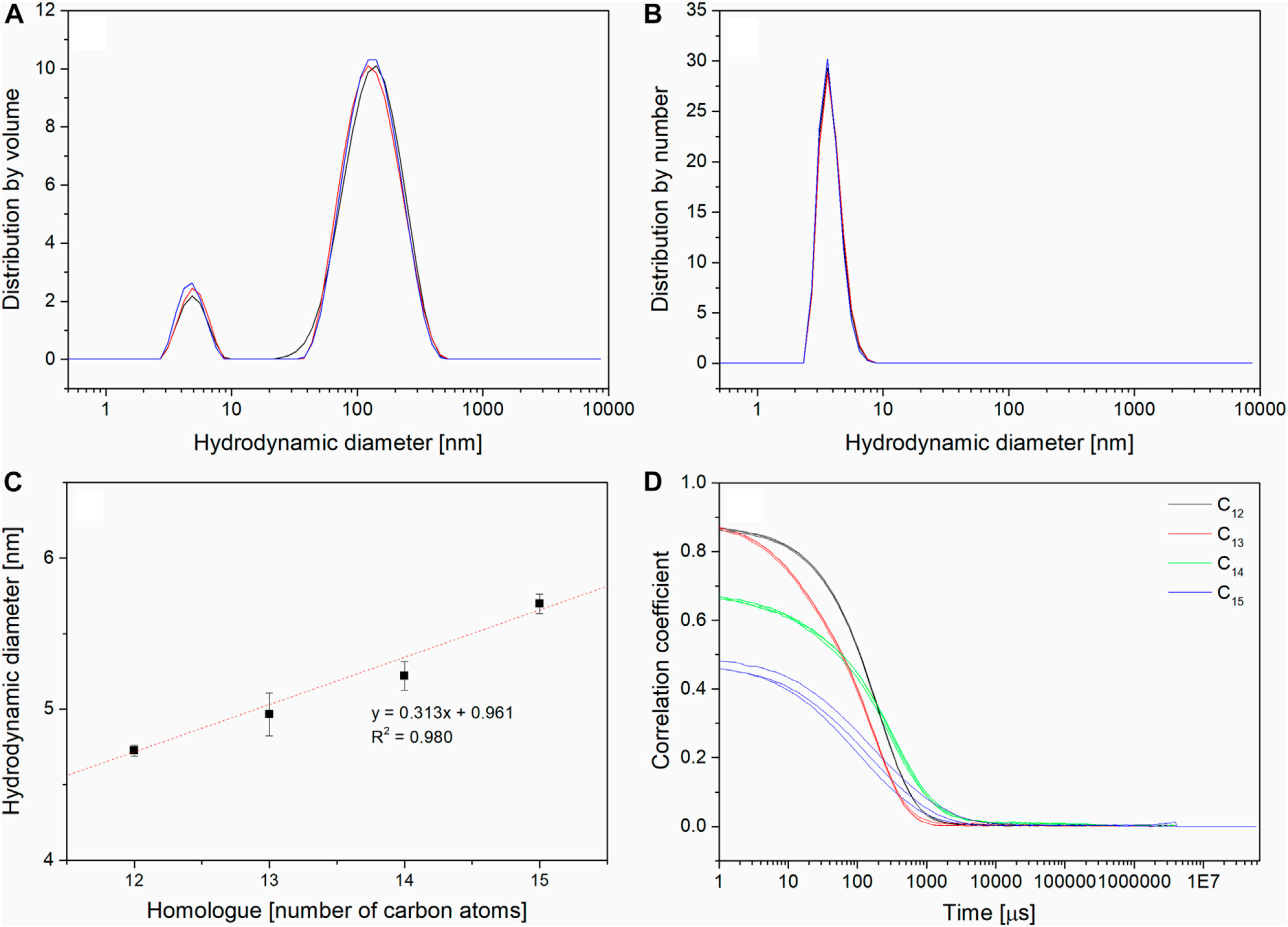
DLS results: **(A)** size distribution in terms of volume for C13 (3 replicates); **(B)** size distribution in terms of number for C13 (3 replicates); **(C)** comparison of homologues’ hydrodynamic diameters; **(D)** comparison of homologues’ correlograms (overlay of 3 measurements for every homologue).

Pure surfactin in form of protonated acids is insoluble in water, so DLS measurements were conducted in 0.05 TRIS×HCl buffer (pH = 8.5) which is typically used to observe single micelles without aggregates (Li, Zou, et al., 2009; Jauregi et al., 2013). Homologues were analyzed in order to determine hydrodynamic diameters of formed micelles. Size distributions by relative intensities and relative number were compared. In the case of all four homologues, the relative intensity distribution shows two populations, a weak peak for sizes 4–6 nm (single micelles) and a three times higher peak for aggregates with over 100 nm (shown on Figures 7A, B with C_13_ as an example). Recorded signal is much stronger for big aggregates than for small ones. The light scattering strongly depends on the volume of particles: the big ones give a strong signal even in small amounts because the radius cubed gives the volume (Abe, 2019) So compared to the distribution by number (where only single micelles are observed), it can be stated that single micelles are the major population of the sample (Han et al., 2008). The measured sizes ranged from 4.7 nm (for C_12_) to 5.7 nm (for C_15_) (Table 4 *exp. DLS*). Similar sizes were reported for SU mixture by other authors (Han et al., 2008; Zou et al., 2010; Jauregi et al., 2013) but there is no available data about pure homologues micellar sizes set together. Comparison of results regarding the homologue chain length showed a linear dependence, where the diameter of the micelles increases with the increase of the number of carbon atoms (Figure 7C). Such observation was expected based on other research done on homologues of surfactants (Gracia et al., 2004; Lewińska et al., 2014). From C_12_ to C_15_, the micelles become less spherical and more oval, which is confirmed by measured correlograms (three for every homologue) showing a decrease in correlation coefficient (CC) with an increase of carbon atom number in the homologue (Figure 7D). So far, the published data on SU mixture micelles provides observation of cylindrical shapes in pH 9.5 which moved to the spherical and ellipsoidal ones after NaCl and CaCl_2_ addition (Knoblich et al., 1995). It shows that the shape of SU’s aggregate is environment-dependent. The presence of complexing cations can promote formation of higher order aggregates. It could explain why in the PBS and NaHCO_3_ large (about 100 nm) aggregates are measured instead of single micelles (Li, Zou, et al., 2009; Liu et al., 2015). Such big aggregates were also observed in other assays as, for example, (Arutchelvi et al., 2014) where the sizes distribution for mixture of surfactins was investigated. Populations of 180 nm and 800 nm sizes were observed and explained with formation of vesicles and large aggregates (Zou et al., 2010). also shows the presence of aggregates different than single micelles described as large fractal ones based on the small angle neutron scattering and freeze-fracture transmission electron microscopy data. Their presence is explained by the hydrogen bonds formation between peptide rings of surfactin.

**TABLE 4:** Properties of examined micellar systems predicted at the DFT theory level: molecular polarity indexes (MPI), minimal (EP_min_) and maximal (EP_max_) values of electrostatic potential, micelle diameters (d_mic_), molecular volumes of monomeric SU (V_mon_), micelle.

		MPI [kcal/mol]	EP_max_ [kcal/mol]	EP_min_ [kcal/mol]	d_mic_ [nm]	V_mon_ [nm^3^]	V_mic_ [nm^3^]	N_agg_
C_12_	*anteiso-*	78.2	1.85	−182.39	3.84	1.33	29.89	22
	*iso-*	77.9	1.85	−182.35	3.86	1.34	30.26	23
	*n-*	77.5	1.82	−182.32	4.12	1.34	36.61	27
	*exp from DLS*	—	—	—	4.7	—	—	—
C_13_	*anteiso-*	77.0	1.78	−182.29	4.12	1.36	36.45	27
	*iso-*	76.9	1.71	−182.24	4.14	1.36	37.29	27
	*n-*	76.6	1.82	−182.33	4.34	1.36	42.68	31
	*exp from DLS*	—	—	—	5.0	—	—	—
C_14_	*anteiso-*	76.1	1.82	−182.27	4.36	1.38	43.57	31
	*iso-*	76.0	1.89	−182.28	4.32	1.38	42.41	31
	*n-*	75.7	1.78	−182.31	4.60	1.39	50.87	37
	*exp from DLS*	—	—	—	5.2	—	—	—
C_15_	*anteiso-*	75.2	1.64	−182.24	4.62	1.41	51.87	37
	*iso-*	75.0	1.67	−182.26	4.62	1.41	51.33	36
	*n-*	74.8	1.80	−182.33	4.82	1.41	58.74	42
	*exp from DLS*	—	—	—	5.7	—	—	—

In order to prove the decrease in spherical shape of single micelles, the correlograms were compared. The value of correlation coefficient in DLS measurement provides information about the quality of the measured data. The lower its value in t_0,_ the lower the correlation of measured scattered light (Goldburg, 1999). An ideal and practically unattainable situation is CC equal 1, but in reality, every measurement with CC above 0.8 is adopted as high-quality data. In homologous series, it can be observed that for C_12_ and C_13_ CC values are good, but for C_14_ the CC value decreases, and for C_15_ it reaches the lowest level. Additionally, for C_12_, C_13_ and C_14_, all three replicates of correlograms are identical, while for C_15_ they vary, which suggests variations in measurements resulting most probably from the change in shape. DLS is a method dedicated for spherical nanoparticles, so every difference in shape causes higher variation of measurement (Pecora, 2000; Stetefeld et al., 2016). Moreover, irregular shapes can cause overestimation of the hydrodynamic diameters due to the stronger light scattering of the bigger dimension. This effect can be additionally enhanced by the hydration shell (Varga et al., 2020). The observation of sphericity disturbance with increasing length of the homologue was supported by TEM observations and the results of DFT calculations.

### 3.5 TEM

**FIGURE 8:**
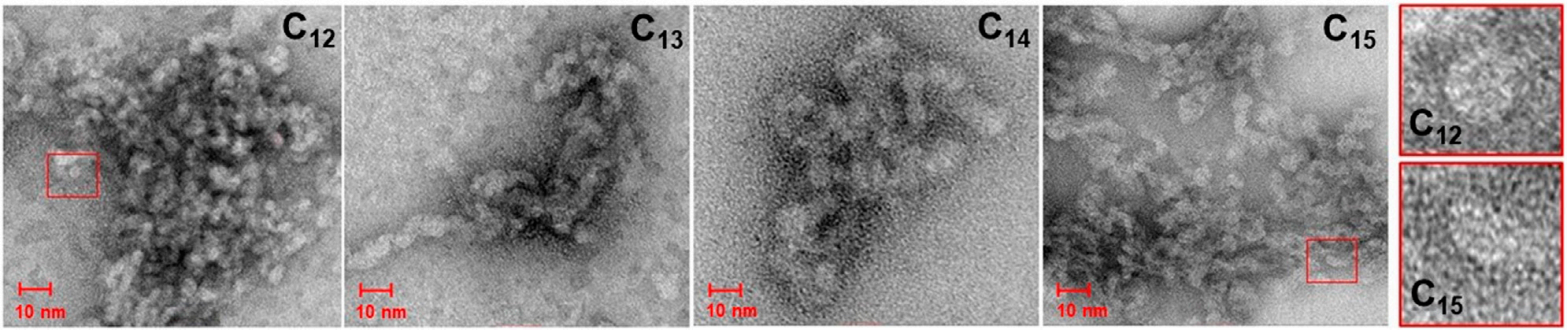
TEM imaging of surfactin homologues micelles and shape comparison.

Transmission electron microscopy was used to investigate the morphology of the obtained micelles (Figure 8). TEM imaging revealed quasi-spherical shapes. A subtle change of shape from spherical to eliptic with increasing length of hydrophobic chain was observed. The observed sizes, after considering the contrast layer, were in reasonable agreement with the DLS measurements. Some differences could have resulted from the different sample preparation processes and different measurement techniques.

The change of spherical to more cylindrical shape of micelles formed by SU homologues with growing hydrophobic tail from C_12_ to C_15_ is most probably due to a combination of three factors: increased hydrophobic interaction between the tails, decrease in *CMC*, and decreased curvature of the micelle surface (formation of lager micelles). The spherical shape of micelles formed by surfactants is a delicate balance between the hydrophobic and hydrophilic properties of the surfactant molecules. As the hydrophobic tail of a surfactant molecule grows from C_12_ to C_15_, the hydrophobic interaction between the tails becomes stronger. In a spherical micelle, the hydrophobic tails are arranged radially around the center of the micelle, with the hydrophilic head groups facing outward. This arrangement maximizes the surface area of the hydrophobic tails that can be shielded from the aqueous environment, while minimizing the surface area of the hydrophilic head groups that are exposed to water. As the hydrophobic tail grows longer, they occupy bigger volumes leading to increased hydrophobic interaction between the tails, which can cause the surfactant molecules to pack more tightly, reducing overall or local curvature of the micelle surface (changing shape and diameter, which has grown from 4.7 to 5.7 nm). Additionally, the increase in the size of the hydrophobic tail leads to a decrease in the critical micelle concentration *CMC* (from 0.35 to 0.08 M). As the *CMC* decreases, the concentration of surfactant molecules in solution increases, contributing to the formation of larger micelles, which may have a more cylindrical shape.

### 3.6 DFT computations

We performed all the DFT computations for the anionic form of SU (deprotonated carboxyl groups of Glu and Asp) because the DLS experiments were performed in TRIS×HCl buffer (pH = 8.5).

All the SU homologues are highly asymmetric systems and have non-zero dipole moment. To illustrate the polarity of SU, we calculated molecular polarity index (MPI), which is a descriptor expressed as (Liu et al., 2021):

$$MPI=\frac{1}{A}\iint_{S}V\left(r\right)|dS$$ (6)

where A and V(r) refer to the area of van der Waals (vdW) surface and the value of EP at a point **r** in space, respectively. In eq. X6, the integration is performed over the whole molecular vdW surface (S). The calculated MPI values of the SU homologues are listed in Table 4 and are found to be in the range 74.8–78.2 kcal/mol. The MPI value decreases with the *β*-hydroxy fatty acid length and is always slightly lower for *anteiso-* and *iso-* than for *n-* isomer. The polarity of SU is high since the predicted MPI values are significantly higher in comparison with the corresponding indexes reported previously for model nonpolar systems, that is 2.6, 6.7, and 8.4 kcal/mol for ethane, ethene, and benzene, respectively (Liu et al., 2021). They evince that the electrostatic interaction between SU and other polar molecules should be fairly strong. However, local polarity in the SU molecules should vary because all heteroatoms are distributed only in the peptide ring. An electrostatic potential (EP) map should be an efficient tool for illustrating the local SU polarity.

**FIGURE 9:**
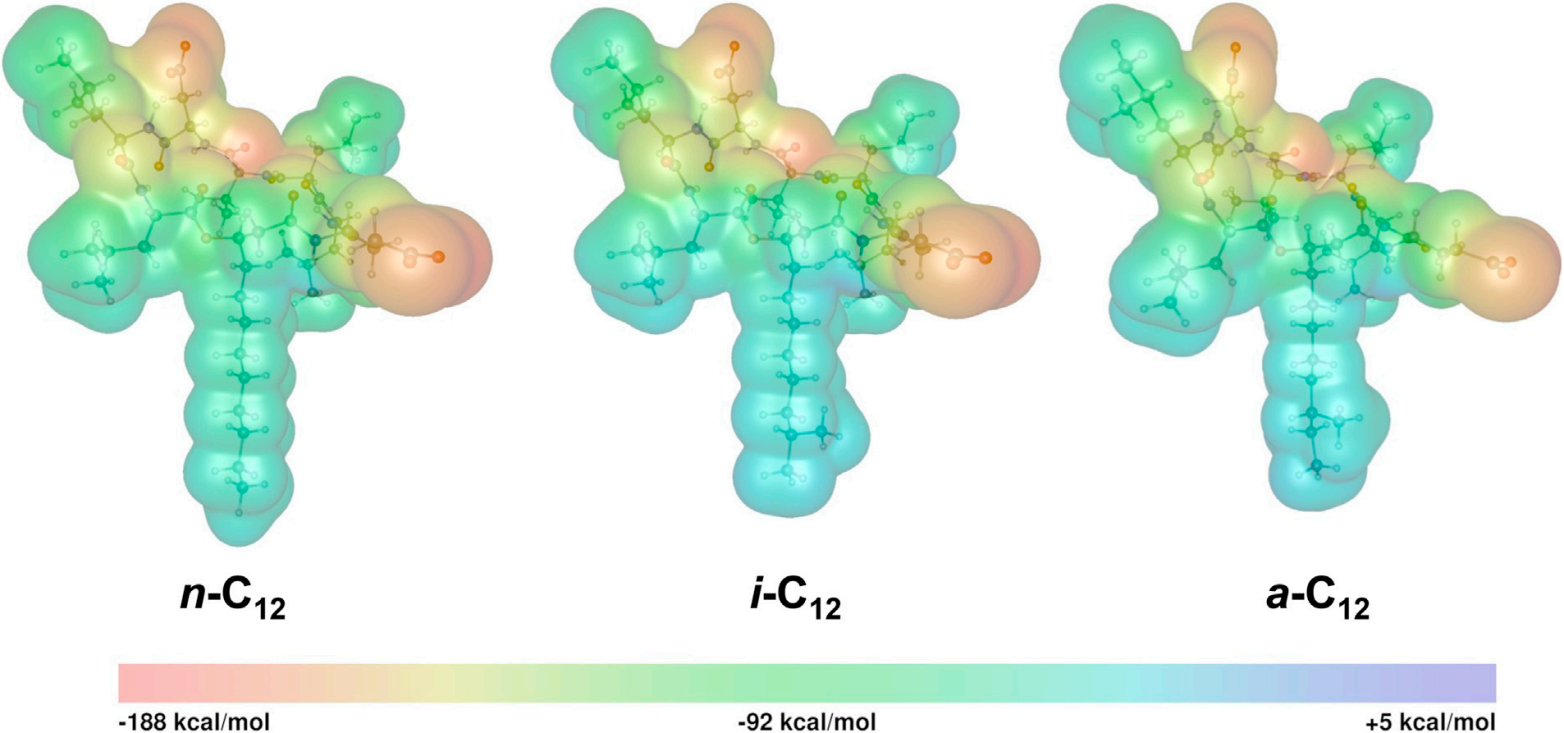
Isosurface maps of the EP for three isomers of C_12_ homologue (isovalue 0.001 au) calculated with the functional B3LYP and basis set def2-TZVP.

The isosurface maps of the EP for three isomers of C_12_ are shown in Figure 9. From these isosurfaces, it can be seen that most regions of SU show negative EP, as expected for an anionic molecule, in contrast to polar neutral molecules for which clear positive and negative zones are expected (Lewińska et al., 2021). Although the EP is generally negative, its distribution is strongly irregular, with the minimal (EP_min_) and maximal (EP_max_) values of ca. −182 and +2 kcal/mol, respectively. The EP_min_ and EP_max_ values are given in Table 4.

In SU, the most negative regions are circular and surround oxygen atoms in the peptide ring. As discussed by Durán-Álvarez et al. (Durán-Álvarez et al., 2016), such a strong charge localization reflects a hard electrostatic nature of atoms being the primary charge carriers in SU. The flow of negative charge to the hydrophobic tails and aliphatic side-chains of amino acid L residues is strongly limited. The lack of significant negative charge on the fatty acid tails shows that, even in its anionic form, SU preserves its amphiphilic structure responsible for its surface and biological activities.

The approximate size of the SU micellar aggregates was estimated using the DFT calculations (Table 4). The micelle diameter (d_mic_) at the DFT level was approximated as the longest distance between two carbon atoms in optimized molecular structures of the SU homologues. The predicted d_mic_ values increase with the hydrophobic chain length from 3.84 nm for *a*-C_12_ to 4.82 nm for *n*-C_15_. Hence, the SU micelle diameter can be directly controlled by the number of carbon atoms in the *β*-hydroxy fatty acid chain. A similar phenomenon was observed, for instance, for the homologous series of fluorinated (Matsuoka et al., 2006; Matsuoka et al., 2007) and di-N-oxide surfactants (Lewińska et al., 2012; Lewińska et al., 2014). Although this rule seems to be taken for granted, several exceptions have been reported. For example, the tendency for the micelle diameter to increase with the number of carbon atoms in the aliphatic chain was not observed for divalent cationic surfactants composed of fluorocarbons and double quaternary ammonium groups (Matsuoka et al., 2011).

The predicted d_mic_ values for all isomers of each homologue are noticeably underestimated compared to the DLS results. However, it is important to acknowledge certain limitations when comparing the results of quantum mechanical calculations with DLS measurements. DLS directly measures hydrodynamic quantities, such as translational and/or rotational diffusion coefficients, which are then related to size and shape through theoretical relationships (Pecora, 2000; Lim et al., 2013). As a result, DLS measurements provide hydrodynamic diameter that can be significantly larger than the geometric particle diameter obtained from DFT predictions, due to the inclusion of possible solvation layers, for instance. It is worth noting that particle diameters determined by DLS are frequently larger than those obtained using other experimental techniques (Lim et al., 2013; Souza et al., 2016). In the case of the SU micelles studied here, which were measured in TRIS×HCl buffer (pH = 8.5), the difference between the DFT and DLS diameter is expected to be further magnified due to the incorporation of relatively large, protonated tris(hydroxymethyl)aminomethane molecules into the layer around the micelles to balance the negative charge of aggregating deprotonated surfactin. Additionally, the discrepancy between theory and experiment may also be influenced by the anisotropic shape of the micelles, as we observed their slightly elliptical shape by TEM imaging (Figure 8).

We attempted to estimate aggregation numbers (N_agg_) at the DFT theory level, assuming a spherical shape of the micellar aggregates, because the divergence from a perfect sphere could not be quantitatively determined. First, the micelle volumes (V_mic_) were calculated from the equation:

$${V_{mic}}^{DFT}=\frac{4}{3}∙\pi ∙{\left(\frac{1}{2}{d_{mic}}^{DFT}\right)}^{3}$$ (7)

leading to the simple expression for N_agg_:

$$N_{agg}=\frac{V_{mic}^{DFT}}{V_{mon}^{DFT}}$$ (8)

Previously, the molecular volumes of the hydrated monomers (V_mon_) were computed using numerical Monte Carlo (MC) integration with a density contour of 0.001 au (Ferreira et al., 2010; Lewińska et al., 2012; Lewińska et al., 2014). Although the high accuracy of this procedure has been demonstrated, in this work we used the Marching Tetrahedra algorithm (Lu and Chen, 2012b) for computation of V_mon_ instead of repeated random sampling of MC methods.

We found that the differences between the molecular structures of the peptide ring are insubstantial between different SU homologues and their isomers. Thus, the calculated d_mic_, V_mon_ and V_mic_ values mainly depend on the fatty acid chain length. As a result, the N_agg_ number is always the highest for the *n*-isomer for each isoform. The number of surfactin molecules building a single micelle is predicted to increase significantly when the fatty acid chain is extended, for instance, from 23 for *i-*C_12_ to 36 for *i-*C_15_, or from 27 for *n-*C_12_ to 42 for *n-*C_15_.

To put this result into perspective, we can compare it with the rise in N_agg_ on the number of carbon atoms in the aliphatic chain reported for two-headed single-chain di-N-oxide surfactants of the C_n_(DAPANO)_2_ and C_n_-MEDA series (Lewińska et al., 2012; Lewińska et al., 2014). For C_n_(DAPANO)_2_ N_agg_ increases from 33 for n = 10 to 91 for n = 16, while for C_n_-MEDA it increases from 49 for n = 10 to 99 for n = 16. The rise in aggregation number for dicephalic di-N-oxide surfactants is significantly greater in comparison with SU. Although these dicephalic surfactants have a relatively large hydrophilic end, they are noticeably smaller and less hydrophilic in comparison with SU. These inimitable structural features of SU result in several unique properties of their aggregate states, such as a lowered aggregate size and aggregation number.

Our theoretical analysis demonstrates that the aggregation numbers for SU micelles are significantly smaller compared to those of typical surfactants, as evidenced by the comparison with C_n_(DAPANO)_2_ and C_n_-MEDA in the preceding paragraph. Therefore, it is crucial to compare these theoretical findings with experimental values obtained from small angle neutron scattering (SANS). Shen et al. investigated the SU mixture with varying *β*-hydroxy fatty acid chains, produced by the strain BBK006 bacteria, and reported an aggregation number of 20 ± 5 (Shen et al., 2009). It is important to note that in the SU mixture used by Shen et al., the dominant homologue was C_13_. Zou et al. studied the [Glu1, Asp5]-C_15_ isoform from *B. subtilis* HSO121 and found the SU aggregation number to be “unbelievably small” (less than 20), which they considered to be similar to the results of Shen et al. (Zou et al., 2010). A direct comparison between theory and the SANS experiments is difficult due to the influence of experimental conditions such as pH or the presence of various homologues in the SU mixture on N_agg_. Nevertheless, our DFT calculations, based on the molecular volumes derived from electron density and the micelle diameters obtained from molecular structures of SU molecules, consistently yield small values for N_agg_. They align well with the experimental results, particularly our prediction of N_agg_ = 27 for *iso*-C_13_, which closely resembles the N_agg_ = 20 ± 5 reported by Shen et al. (Shen et al., 2009).

### 3.7 Contact angle assay

**FIGURE 10:**
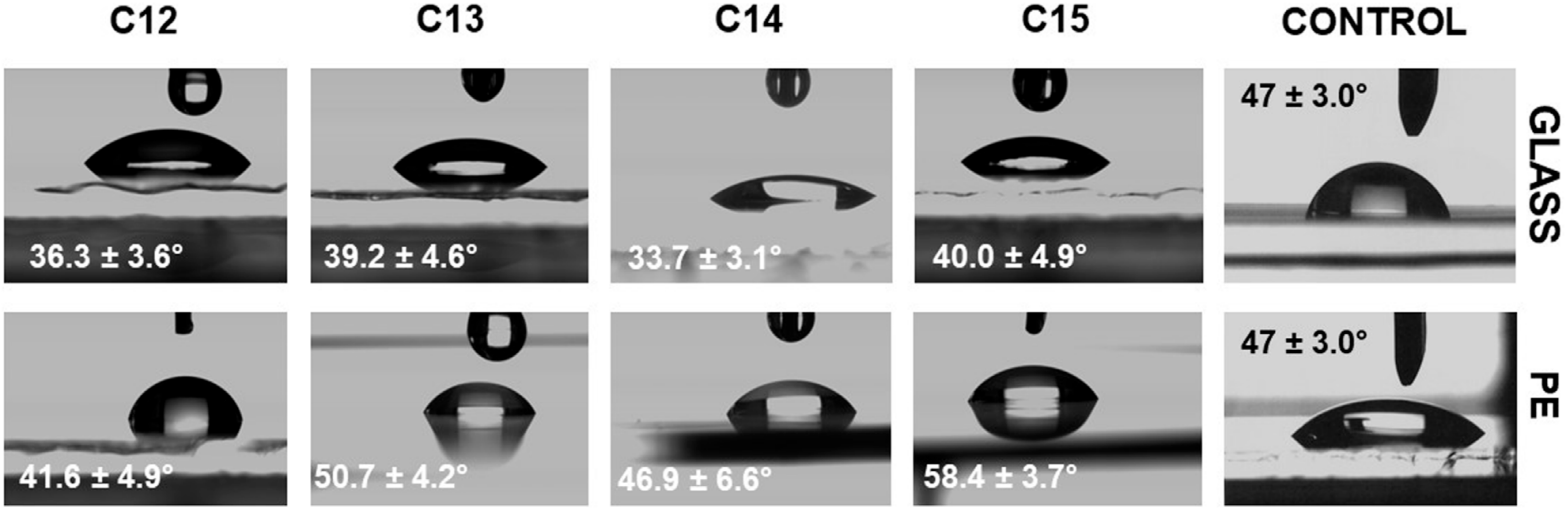
Contact angle assay results.

The wettability of the solution in relation to the surface with which it is in contact can be determined by measurement of the contact angles (Luner et al., 1996; Zhang et al., 2010). Contact angle is the angle between the contact plane and the tangent line to the droplet derived from the contact point of the three phases, solid, liquid and gas. If the measured value is lower than 90°, it means that the analyzed liquid prefers to develop the surface of contact with a solid, so it has good wetting properties. In the opposite situation, when the angle is higher than 90°, it means that the solution avoids contact with the surface and wetting is weak (Mohammadi et al., 2004; Lamour et al., 2010). For the contact angle assay, two types of surfaces were used, glass (as the hydrophilic one) and polyethylene–PE (as the hydrophobic one) and the results are shown in Figure 10. Concentration of 0.11 mg/mL of SU’s homologues was tested in 0.05 M TRIS×HCl (pH = 8.5), and the buffer was measured as the control sample. For PE, the contact angle was reduced to about 50° from 71.4° depending on homologue. This means that SU improves wettability by 30%, which is comparable with sodium dodecyl sulphate, whose minimum contact angle in a water-polyethylene system is between 50° and 60° (Maſko et al., 2016). The strongest effect was recorded for C_14_ resulting in 46.9°, and C_13_ (50.7°), the weakest for C_12_ (74.8°). C_15_ (58.4°) was between them. In the case of glass, the pattern was similar–the strongest effect was measured for C_14_ (33.7°), while C_13_ and C_15_ were not significantly different (39.2° and 40.0°) and C12 (51.2°) was the weakest one. SU’s wetting ability in contact with glass can be compared to the Triton-TX165, whose water solution gives minimum contact angle equal to 36° (Szymczyk and Jańczuk, 2008). One would expect that the contact angle values ​​would decrease with increasing force of the surface tension reduction of the homologues, and therefore in the order C_12_ < C_13_ < C_14_ < C_15_. Indeed, the weakest wettability is observed for the C_12_ homologue, but in the case of C_13_, C_14_ and C_15_, the recorded differences are small. C_14_ shows the best wetting ability, C_13_ and C_15_ slightly lower. This is consistent with other literature reports, where, for example, the wettability angles for sodium dodecyl sulphate and sodium hexadecyl sulphate (which differ in as many as four carbon atoms) were compared and the same insignificant differences were shown (Zdziennicka et al., 2003). An interesting fact is that the quantitative distribution of homologues in a natural mixture of surfactin correlates with the strength of wetting ability. It can be seen from Figure 1A that the most abundant homologue is C_14_, then C_15_ and C_13_, whose quantities are similar. C_12_, having the weakest wetting properties, is the least abundant. One of the natural functions of SU is an improvement of biofilm formation, which is important in the expansion of *B. subtilis* in the environment (Bais et al., 2004; Leclère et al., 2006; López et al., 2009) High wetting ability can be a crucial parameter for the growth of biofilm and gliding, so the C_14_ homologue as the most effective agent is produced in the highest quantities. SU is produced by bacteria species, including some strains known to exhibit gliding motility. Gliding motility is a form of bacterial movement that allows cells to move smoothly and rapidly along a solid surface without the use of flagella. The mechanism of gliding is still not completely understood. One of the proposed mechanisms is the “slime propulsion” model, which suggests that gliding motility is driven by the flow of a slimy extracellular matrix that is produced by the bacteria where contact angle and surface tension may be a partial driving force of the flow.

The results indicate that SU can be effectively used as a wetting agent, with its mixture showing similar efficacy to that of the pure homologues when applied immediately after microbial production. Moreover, the low toxicity, biodegradability, and biocompatibility of SU (de Oliveira et al., 2017) make it a promising candidate for various applications, such as the removal of hydrophobic environmental pollutants (Mulligan, 2005; Wang et al., 2020; Meena et al., 2021).

### 3.8 Emulsification indexes (E_24_)

**FIGURE 11:**
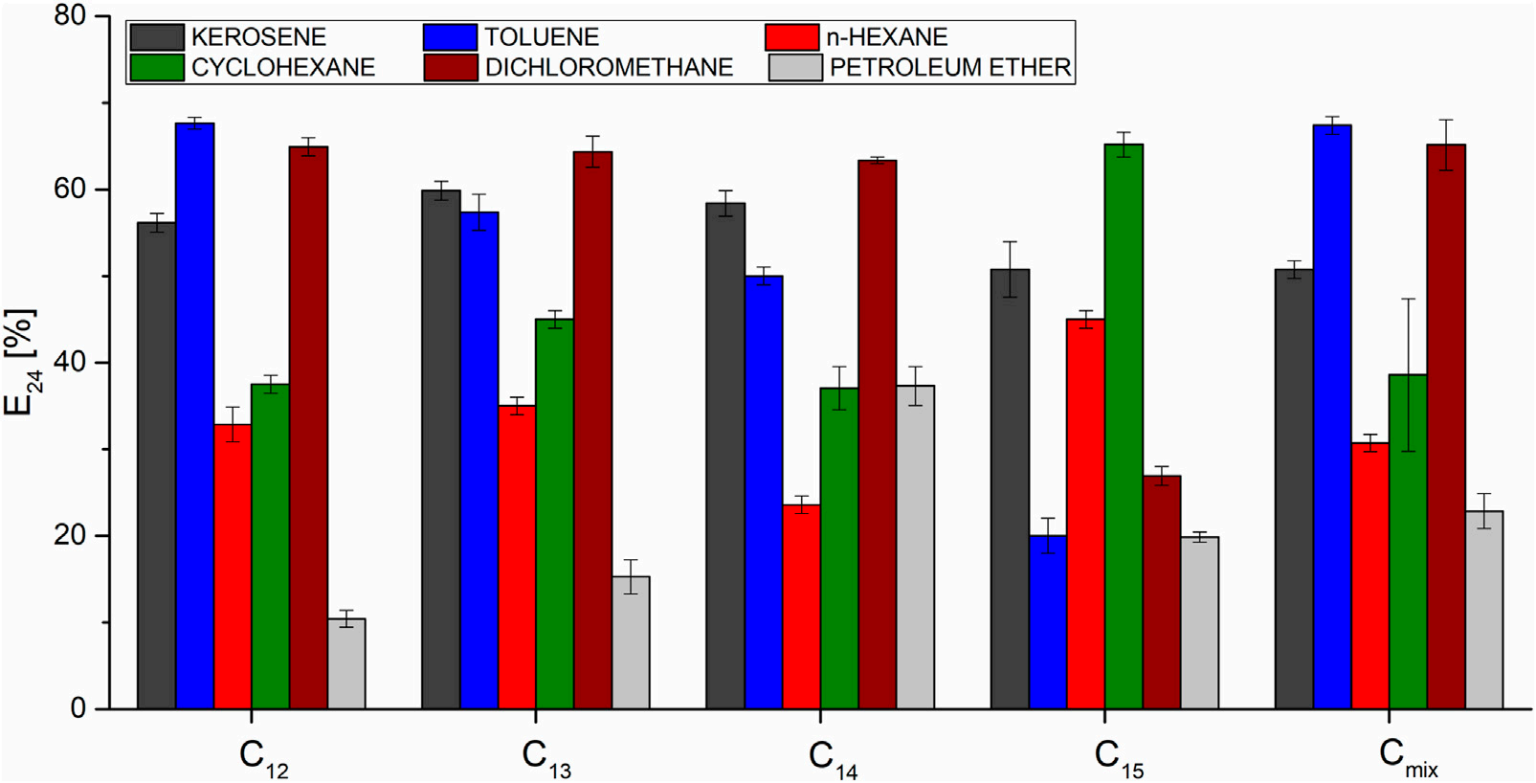
Emulsification indexes determined for SU homologues.

In order to compare emulsification ability of particular homologues, emulsification indexes were determined for different hydrophobic phases. The tested substances were kerosene, toluene, *n*-hexane, cyclohexane, dichloromethane and petroleum ether. Results obtained for the homologues (Figure 11) completely disagreed with their measured surface tension reduction abilities, which is consistent with previous research done on biosurfactants (Cooper and Goldenberg, 1987; Menezes Bento et al., 2005). In addition, the one strongest homologue was not observed. The intensity of emulsification was different for the tested hydrophobic phases and, for example, C_14_ was the most efficient emulsifier in the case of petroleum ether (37.3%) but the worst one in the case of *n*-hexane (23.6%) and cyclohexane (27.5%). C_15_ was the best emulsifier for *n*-hexane (45.0%) and cyclohexane (65.2%), and worst in the case of kerosene (50.8%), toluene (20.0%) and dichloromethane (26.9%). The best overall emulsification strength of SU was observed for kerosene (about 60%), toluene, cyclohexane and dichloromethane (about 70%). Emulsification indexes of *n*-hexane and petroleum ether were the worst. E_24_ determined for the native mixture of homologues (non-separated sample) was not the average value of its ingredients. This means that contributions of particular homologues to the overall effect is not equal. However, several dependencies have been observed. Petroleum ether is a fraction of oil distilled in the temperature range of 40°C–60°C and forms the mixture of pentane and hexane isomers. In relation to this substance, C_15_ shows a much higher EI24 than the other homologues, while showing a much lower ability to homogenize toluene. This means that the length of the homologue may be important for the homogenization of aliphatic and aromatic compounds - shorter ones are more effective against aromatic hydrocarbons. Kerosene, which is distilled from oil at temperatures of 170°C–250°C, contains mainly C_12_-C_15_ alkanes and is much more effectively emulsified by all surfactin samples than petroleum ether. This means that surfactin has better emulsifying properties than alkanes with long hydrocarbon chains. Taking into account its structure, it makes sense, because the interaction with non-polar phases is mediated by hydrophobic chains of *β*-hydroxy fatty acids, the lengths of which are closer to those of kerosene than petroleum ether. Short hydrophobic chains are present in the amino acid ring, but a partial electrostatic charge bearing peptide bond and D and E residues prevent them from interacting with hydrophobic substances. There is also a noticeable difference between the group C_12_-C_14_ and C_15_ in the E_24_ for dichloromethane, shorter homologues show a higher efficiency against halogen derivatives. In addition, for all homologues, a better ability to emulsify cyclohexane than hexane is observed, which means that, in general, surfactin is more likely to form an emulsion from cyclic hydrocarbons. The known fact is that SU homologue composition strongly depends on the environmental factors (Bartal et al., 2018). Emulsification of nonpolar substances is the way to make them more bioavailable for bacteria. It is possible that depending on the surrounding’s properties, *Bacillus* produces homologues with different compositions in order to emulsify hydrophobic materials since they have various emulsifying potentials.

## 4 Conclusion

Flash chromatography has proven to be the most effective method for separating SU homologues. Using this technique, we obtained C_13_, C_14_, and C_15_ homologues with yields of over 100 mg from a single injection. We also isolated small amounts of C_12_ for comparative analysis. LC-MS and GC-MS analyses confirmed that the obtained compound samples were homologue pairs. Using Gibbs isotherms, we compared the surface tension reduction ability and *CMC* values, and found that C_15_ was the strongest surfactant molecule, with increasing surface activity observed with increasing homologue length. TEM, DLS, and DFT techniques were used to describe the micellization behavior, revealing that the less spherical the micelle, the more carbon atoms present in the homologue, with hydrodynamic diameters ranging from 4.7 to 5.7 nm between C_12_ and C_15_. Computational data obtained with DFT showed that SU has a very low aggregation number. Based on isosurface maps, we concluded that SU molecules have a large polar surface area and a relatively small hydrophobic moiety, suggesting that SU, when aggregating, contributes more to the formation of the micelle surface than to its internal volume.

The usefulness of surfactants relates to micelle formation, as these aggregates are responsible for the washing effect. A low aggregation number means that fewer molecules are necessary to form a micelle, and thus, a surfactant with a lower aggregation number at the same concentration is a more powerful washing agent compared to one with a high aggregation number. To describe the functional properties of the homologues, we investigated contact angles and emulsification indexes, finding that the strength of surface reduction did not consistently correlate with wetting ability or emulsification capability. C_14_ was the most abundant homologue in the natural mixture and the best wetting agent according to our contact angle assay results. This homologue is likely produced in abundance due to its positive impact on *Bacillus* biofilm formation. However, the contribution to gliding motility is not the only function of SU. *Bacillus* can secrete SU into the environment to emulsify hydrophobic substances and make them more bioavailable. From the E_24_ analysis, we observed that there is no consistent rule among homologues in emulsification strength. The peptide chain enables a multitude of interactions with other compounds, and different homologues have various emulsifying efficiencies with tested substances, indicating that an SU mixture containing small amounts of various homologues improves *Bacillus*’ ability to cope with environmental conditions due to better emulsifying ability than a single structure.

This point of view allows us to understand the significance of SU for the producing organism better. It appears that SU is not only one of the strongest known biosurfactants with high utility value but also a powerful tool for the succession of bacteria in its environment. The published methodology, based on SU, can be applied to other similar structures of cyclic lipopeptides, allowing for a better understanding of their structure-properties relationship.

## Data Availability

The raw data supporting the conclusion of this article will be made available by the authors, without undue reservation.
