# Supplementary material for: Formation and structural features of micelles formed by surfactin homologues
Source: Front Bioeng Biotechnol. 2023 Jul 7;11:1211319. doi: 10.3389/fbioe.2023.1211319 (PMC10360134; doi:10.3389/fbioe.2023.1211319)
Supplement: Supplementary file 1 [file Table1.docx]

**Supplementary Materials**

**Formation and structural features of micelles formed by surfactin homologues**

**Michał Bochynek^1,4^, Agnieszka Lewińska^2,5*^, Maciej Witwicki^2^, Agnieszka Dębczak^3^, Marcin Łukaszewicz^1,4*^**

1. Department of Biotransformation, Faculty of Biotechnology, University of Wroclaw, Wroclaw, Poland
2. Faculty of Chemistry, University of Wroclaw, Wroclaw, Poland
3. Łukasiewicz Research Network—New Chemical Syntheses Institute, Puławy, Poland
4. InventionBio S.A., Bydgoszcz, Poland
5. OnlyBio S.A., Bydgoszcz, Poland

*** Correspondence:**[agnieszka.lewinska@uwr.edu.pl](mailto:agnieszka.lewinska@uwr.edu.pl)

[marcin.lukaszewicz@uwr.edu.pl](mailto:marcin.lukaszewicz@uwr.edu.pl)

Keywords: surfactin, *Bacillus subtilis*, homologues, micelles, adsorption.

Table 1. Summarized results for surfactin homologues characterization.

| **Homologue** | | **C_12_** | **C_13_** | **C_14_** | **C_15_** |
| --- | --- | --- | --- | --- | --- |
| **Peptide ring sequence** | | ELLVDLL | ELLVDLL | ELLVDLL | ELLVDLL |
| ***β*-hydroxy fatty acid branching** | | *n-* and *iso-* | *iso-* and *anteiso-* | *n-* and *iso-* | *iso-* and *anteiso -* |
| **Molecular mass [Da]** | | 993.5 | 1007.6 | 1021.6 | 1035.6 |
| ***γ_min_* [mN/m]** | | 32.2 | 30.0 | 28.5 | 27.6 |
| **10^6^ *Γ_∞_* [mol/m^2^]** | | 2.57 | 2.60 | 2.50 | 2.45 |
| **10^20^ *A*_min_ [m^2^]** | | 149 | 147 | 153 | 156 |
| **p*C*_20_ [M]** | | 2.43*10^-4^ | 9,13*10^-5^ | 3.11*10^-5^ | 9.79*10^-6^ |
| **-Δ*G*^0^_ads_ [kJ/mol]** | | 20.41 | 22.81 | 25.43 | 28.29 |
| ***CMC* [mmol/L]** | | 0.35 | 0.24 | 0.17 | 0.08 |
| **d_mic_ (DLS) [nm]** | | 4.7 | 5.0 | 5.2 | 5.7 |
| **d_mic_ (DFT) [nm]** | *anteiso-*  *iso-*  *n-* | 3.84  3.86  4.12 | 4.12  4.14  4.34 | 4.36  4.32  4.60 | 4.62  4.62  4.82 |
| **V_mon_ (DFT) [nm^3^]** | *anteiso-*  *iso-*  *n-* | 1.33  1.34  1.34 | 1.36  1.36  1.36 | 1.38  1.38  1.39 | 1.41  1.41  1.41 |
| **N_agg_ (DFT)** | *anteiso-*  *iso-*  *n-* | 22  23  27 | 27  27  31 | 31  31  37 | 37  36  42 |
| **Contact angle [°]** | (glass) | 51.2 +/- 2.4 | 39.2 +/- 4.6 | 33.7 +/- 3.1 | 40.0 +/- 4.9 |
|  | (polyethylene) | 74.8 +/- 4.9 | 50.7 +/- 4.2 | 46.9 +/- 6.6 | 58.4 +/- 3.7 |
| **E_24_ [%]** | (kerosene) | 56.1 +/- 1.1 | 59.9 +/- 1.1 | 58.4 +/- 1.5 | 50.8 +/- 1.0 |
|  | (toluene) | 67.6 +/- 0.7 | 57.4 +/- 2.1 | 50.0 +/- 1.0 | 20.0 +/- 2.0 |
|  | (*n*-hexane) | 32.9 +/- 2.0 | 35.0 +/- 1.0 | 23.6 +/- 1.0 | 45.0 +/- 1.0 |
|  | (cyclohexane) | 37.5 +/- 1.0 | 45.0 +/- 1.0 | 37.1 +/- 2.5 | 65.2 +/- 1.4 |
|  | (dichloromethane) | 65.0 +/- 1.1 | 64.4 +/- 1.8 | 63.4 +/- 1.1 | 26.9 +/- 1.1 |
|  | (petroleum ether) | 10.4 +/- 1.0 | 15.3 +/- 2.0 | 37.3 +/- 2.2 | 19.9 +/- 0.6 |
